# Downregulation of the Autism Spectrum Disorder Gene *Shank2* Decreases Bone Mass in Male Mice

**DOI:** 10.1002/jbm4.10711

**Published:** 2022-12-15

**Authors:** Mubashir Ahmad, Nadine Stirmlinger, Irfana Jan, Ulrich Stifel, Sooyeon Lee, Marcel Weingandt, Ulrike Kelp, Jürgen Bockmann, Anita Ignatius, Tobias M. Böckers, Jan Tuckermann

**Affiliations:** ^1^ Institute of Comparative Molecular Endocrinology (CME) Ulm University Ulm Germany; ^2^ Institute for Anatomy and Cell Biology Ulm University Ulm Germany; ^3^ Institute of Orthopaedic Research and Biomechanics Ulm University Ulm Germany

**Keywords:** BONE MASS, OSTEOBLAST DIFFERENTIATION, SHANK2

## Abstract

Mutations of the postsynaptic scaffold protein Shank2 lead to autism spectrum disorders (ASD). These patients frequently suffer from higher fracture risk. Here, we investigated whether Shank2 directly regulates bone mass. We show that Shank2 is expressed in bone and that Shank2 levels are increased during osteoblastogenesis. Knockdown of Shank2 by siRNA targeting the encoding regions for *PDZ* and *SAM* domain inhibits osteoblastogenesis of primary murine calvarial osteoblasts. Shank2 knockout mice (*Shank2* ^−/−^) have a decreased bone mass due to reduced osteoblastogenesis and bone formation, whereas bone resorption remains unaffected. Induced pluripotent stem cells (iPSCs)‐derived osteoblasts from a loss‐of‐function Shank2 mutation in a patient showed a significantly reduced osteoblast differentiation potential. Moreover, silencing of known Shank2 interacting proteins revealed that a majority of them promote osteoblast differentiation. From this we conclude that Shank2 and interacting proteins known from the central nervous system are decisive regulators in osteoblast differentiation. © 2022 The Authors. *JBMR Plus* published by Wiley Periodicals LLC on behalf of American Society for Bone and Mineral Research.

## Graphical abstract

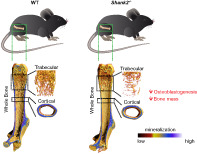

Here we show that loss of function of the postsynaptic scaffold protein Shank2 in mice is accompanied with decreased osteoblastogenesis, which subsequently reduced trabecular bone mass. Our work suggests that the higher fracture risk in patients with autism spectrum disorders (ASD) due to loss of Shank protein expression depends at least in part on the role of Shank2 in bone.

## Introduction

The regulation of osteoblastogenesis, essential for bone formation, is still not completely understood. To tackle bone diseases such as osteoporosis, a profound understanding of osteoblastogenesis is of utmost importance. In recent years, genes previously known to have a particular function in the brain turned out to be important signaling molecules in bone. The semaphorins and the slit/robo genes are prominent examples.^(^ ^1^, ^2^, ^3^, ^4^, ^5^, ^6^, ^7^, ^8^, ^9^ ^)^

Intriguingly, lower bone mineral density (BMD) and increased risk of fractures have been observed in both children and young adults with autism spectrum disorder syndromes (ASDs).^(^ ^10^, ^11^, ^12^ ^)^ Mutations in SH3 and multiple Ankyrin repeat domain protein 2 (Shank2), originally identified as cortactin‐binding protein 1 (CortBP1) or proline rich synapse associated protein 1 (ProSAP1), have been reported to be associated with ASDs.^(^ ^13^ ^)^ Shank2 belongs to a family of proteins (Shank1–3) that are all known to be involved in the pathology of neuropsychiatric diseases. These proteins are highly enriched in the postsynaptic density (PSD) of excitatory synapses.^(^ ^14^, ^15^ ^)^ PSDs are electron‐dense structures containing specific postsynaptic protein complexes that organize the localization and function of receptors, cell adhesion molecules, and signaling cascades.^(^ ^16^, ^17^, ^18^, ^19^ ^)^

Shank proteins contain multiple sites for protein–protein interaction, including ankyrin repeats, an SH3 domain, a PDZ domain, a long proline‐rich region, and a SAM domain.^(^ ^20^, ^21^ ^)^ Over the years, several interacting partners for most of these domains have been identified, such as somatostatin receptor subtype 2 (SSTR2), G protein‐coupled CL1, a guanine nucleotide exchange factor beta PIX, cystic fibrosis transmembrane conductance regulator (CFTR), F‐actin binding protein Abp1, glutamate receptor (GluR) delta2, phospholipase C‐beta3, atypical protein kinase C (aPKC), and SAP90/PSD‐95‐associated protein/guanylate kinase‐associated protein (SAPAP/GKAP) family, in order to regulate several biological processes.^(^ ^22^, ^23^, ^24^, ^25^, ^26^, ^27^, ^28^, ^29^, ^30^ ^)^

Mutations or genetic deletion of the *Shank2* gene are causative for several neuropsychiatric disorders such as schizophrenia (SCZ), bipolar disorder, ASD, and intellectual disability.^(^ ^31^, ^32^, ^33^, ^34^, ^35^ ^)^ Interestingly, core symptoms of these disorders are also found in animal models for Shank2 deficiency. In *Shank2* knockout (*Shank2* ^*−/−*^) mice, reduced communication and altered social interaction were shown to be a consequence of decreased function of N‐methyl‐D‐Aspartate (NMDA) glutamate receptors (NMDAR).^(^ ^36^, ^37^ ^)^ Furthermore, *Shank2* ^*−/−*^ mice showed reduced NMDA‐induced nociceptive responses and spinal NMDA receptor‐mediated pain.^(^ ^38^, ^39^ ^)^ Interestingly, direct stimulation of NMDARs with D‐cycloserine has normalized NMDAR function and improved social interaction in *Shank2* knockout mice.^(^ ^40^ ^)^ In addition, early correction of NMDAR dysfunction in *Shank2* ^*−/−*^ mice exhibited a long‐lasting effect of preventing autistic‐like social behaviors from developing at later stages.^(^ ^41^ ^)^

Although *Shank2* is well characterized in neurobiology, very few studies have reported that *Shank2* is expressed in other non‐neuronal cells where it may fulfill similar functions as in neurons, such as kidney, liver, endocrine cells, pancreas, and thymus.^(^ ^42^, ^43^, ^44^, ^45^ ^)^ Interestingly, a recent study reported that depletion of *SHANK2* impaired osteo/dentinogenic differentiation of human stem cells from apical papilla (SCAPs).^(^ ^46^ ^)^ However, the study suggests that the impact of *SHANK2* downregulation was more specific to differentiation markers of odontoblast lineage rather than osteoblast lineage.^(^ ^46^ ^)^

In this study, we describe the role of *Shank2* in osteoblast differentiation and skeletal homeostasis in vitro and in vivo, respectively. We showed that RNAi‐mediated *Shank2* loss‐of‐function decreased osteoblast differentiation and mineralization. We demonstrated that mice lacking *Shank2* exhibit a decrease in bone mass and osteoblast numbers, which confirms that *Shank2* is a positive regulator of bone formation. We further revealed that loss‐of‐function mutation in *SHANK2* gene in human induced pluripotent stem cells (iPSCs) decreases osteoblast differentiation. These results provide genetic evidence that *Shank2,* in addition to its known function, is an important regulator of osteoblast differentiation and bone mass.

## Materials and Methods

### Mice

The engineering of global *Shank2* ^−/−^ mice has been previously described.^(^ ^36^ ^)^ The mice were housed in groups under controlled standard conditions (diurnal lighting conditions with food and water available *ad libitum*) in a pathogen‐free animal facility at Ulm University. Wild‐type (WT) and Shank2 knockout (*Shank2* ^*−/−*^) 13‐week‐old male littermates were selected for the experiments. The mice were backcrossed to a C57BL/6 background. All experimental procedures were approved by the Regierungspräsidium in Tübingen, Germany (under license no. o.103).

### Cell culture

Primary murine calvarial osteoblasts were isolated from neonatal mouse calvariae of 2‐ to 5‐day‐old pups, as previously described.^(^ ^47^ ^)^ Briefly, the calvariae were isolated in 1 mL digestion solution (0.2% w/v each of collagenase A [cat. 11088793001; Roche, Mannheim, Germany] and dispase II [cat. 04942078001; Roche]) and incubated (37°C; 10 minutes; 700 rpm). The digestion was performed five times, and all but the first supernatant were collected in 15 mL falcons containing 500 μL fetal bovine serum (FBS) (cat. A15‐101; GE Healthcare, Chicago, IL, USA). The collected supernatant was centrifuged (1500 rpm; 5 minutes; room temperature [RT]), and the pellet was resuspended in 3 mL of growth medium and seeded in a 9.6 cm^2^ (6‐well plate). After overnight incubation (37°C; 5% CO_2_), the medium was replaced with a fresh growth medium. The experiments were performed at an 80% confluency.

The primary cells and MC3T3‐E1 cell line were cultured and maintained in α‐MEM medium (cat. 41061037; Thermo Fisher Scientific, Waltham, MA, USA) supplemented with 10% FBS and 1% penicillin/streptomycin (cat. P0781; Sigma‐Aldrich, St. Louis, MO, USA).

The ethical approval for human iPSCs was obtained from the ethical commission of Ulm University (265/12 and 208/16). Human iPSCs were reprogrammed from patient and control keratinocytes as previously described.^(^ ^48^ ^)^ Briefly, human iPSCs were cultured on Matrigel (cat. 354277; Corning, Corning, NY, USA) coated plates in mTeSR1 medium (cat. 05850; StemCell Technologies, Vancouver, Canada). The spontaneously differentiated or dead cells were removed mechanically with pipette tips, followed by a washing step with DMEM‐F12 (cat. 31330–028; Gibco, Thermo Fisher Scientific). Upon reaching 80% confluency or large colony size, the cells were detached using dispase (diluted 1:5 in DMEM‐F12) and a cell scraper. Consequently, the cells were resuspended in mTeSR1 medium and plated into new micro‐dishes (cat. 81156; Ibidi, Grafelfing, Germany).

### Isolation of primary osteoblasts from mouse long bones

The primary osteoblasts were isolated from long bones as previously described.^(^ ^49^ ^)^ Briefly, the long bones (femur, tibia, humerus, radius) were isolated from 4 wild‐type (WT) and 4 *Shank2* ^*−/−*^ 8‐week‐old mice. The bones were transferred to the complete medium (α‐MEM containing 15% FBS; 1% penicillin/streptomycin; and 1% L‐glutamine), and cleared off the soft tissue. The epiphysis were cut and the bones were transferred into 0.2 mL eppendorf tubes containing a hole at the bottom placed in 1 mL eppendorf tubes. Subsequently, the tubes were centrifuged (14,674 *g*; 1 minute; RT) to get rid of the bone marrow. This was followed by transferring bones in 56 cm^2^ culture dishes (10 cm dishes) containing PBS with 1% penicillin/streptomycin. The bones were cleared off from any unwanted material and washed with PBS. Next, the bones were cut into small pieces of approximately 1 to 2 mm^2^ using scissors, washed thrice with PBS, and transferred to 10 mL Collagenase IV (1 mg/ml) (cat. C5138; Sigma) (dissolved in α‐MEM medium), and incubated (37°C; 1 hour) in a shaking water bath. After incubation, the bones were rinsed twice with PBS and once with complete medium. The bone pieces from each mouse were transferred to 56 cm^2^ culture dishes containing 10 mL complete medium. The medium was replaced every third day with fresh medium for 4 weeks. To obtain more cells, the bone pieces were gently washed out with PBS and the cells from each mouse were trypsinized and seeded in two 175 cm^2^ flasks (T175). After reaching 80% confluency, the cells were used for experiments.

### Osteoblast differentiation of murine primary osteoblasts isolated from calvaria and long bones

After 48 hours of seeding (primary calvarial osteoblasts: 1.2 × 10^4^ cells/cm^2^; primary osteoblasts: 2 × 10^4^ cells/cm^2^), the culture medium was replaced with osteogenic induction medium (α‐MEM culture medium containing 100 μg/mL (+)‐sodium L‐ascorbate (cat. A4034; Sigma) and 5 mM ß‐glycerophosphate (cat. G9422; Sigma) to the primary osteoblasts isolated from calvaria and long bones). The osteogenic induction medium was refreshed every third day until the termination of the experiment.

### Differentiation of human iPSCs into osteoblasts

To start a differentiation of human iPSCs into osteoblasts, two densely grown wells were seeded onto six μ‐dishes in 1.5 mL mTeSR1. As soon as the μ‐dishes were densely filled with middle‐sized colonies, differentiation was initiated using the following scheme as previously shown.^(^ ^50^ ^)^ All media were generated by supplementing osteoblast basal medium (OBM) (43.5% DMEM/F12; 43.5% neurobasal (cat. 21103–049; Invitrogen, Carlsbad, CA, USA); 10% FBS (cat. 10500; Gibco, Thermo Fisher Scientific); 1% antibiotic‐antimycotic (cat. 1524006; Invitrogen); 1% N2 supplement (cat. 17502–048; Gibco, Thermo Fisher Scientific); 1% B27 supplement (cat. 17504–044; Gibco, Thermo Fisher Scientific). Days 0–5 (mesoderm differentiation induction): human iPSC were washed with 1 mL of PBS + MgCl_2_/+CaCl_2_, then the OBM‐0 (10 mL OBM supplemented with 25 μM CHIR99021 (cat. 72054; StemCell Technologies); 5 μM cyclopamine (cat. BML‐GR334; Enzo Life Sciences, Farmingdale, NY, USA); 1:1000 Rho‐associated kinase (ROCK)‐inhibitor (cat. Y‐27632; Selleckchem, Houston, TX, USA) medium was added. Medium was changed to OBM‐1 (10 mL OBM supplemented with 30 μM CHIR99021; 5 μM cyclopamine; 1:1000 ROCK‐inhibitor) on day 1. Subsequently, OBM‐1 was changed daily, always after the cells were washed with PBS + MgCl_2_/+CaCl_2_ to remove dead cells. The medium contained ROCK inhibitor to prevent cell death. Days 5–19 (osteoblast differentiation induction): OBM‐1 medium was removed completely, and cells were washed twice with 1 mL of PBS + MgCl_2_/+CaCl_2_. Afterward, 1.5 mL OBM‐2 (10 mL OBM supplemented with 10 mM β‐glycerophosphate; 50 μg/mL ascorbic acid; 0.1 μM dexamethasone (cat. D4902‐25MG; Sigma); 1 μM smoothened agonist (SAG) (cat. 566660; Calbiochem, MilliporeSigma, Burlington, MA, USA); 1 μM TH (cat. M3085; Tokyo Chemical Industry, Tokyo, Japan) containing osteoblast inducers were added. Every 2 days, the cells were washed once with PBS + MgCl_2_/+CaCl_2_ and 1.5 mL of new OBM‐2 was added. Days 19–23 (osteoblast maturation): The old medium was removed completely, and the cells were washed with PBS + MgCl_2_/+CaCl_2_ until no dead cells were visible. Then, 1.5 mL of OBM‐3 (= OBM‐2 without SAG and TH) were added. Every 2 days, the cells were washed once with PBS + MgCl_2_/+CaCl_2_ and 1.5 mL new OBM‐3 was added. At day 23, mature osteoblasts were obtained and used for further experiments.

### Micro‐CT analysis

The high‐performance Bruker (Kontich, Belgium) Skyscan 1176 micro‐CT scanner (version 1.1) acquired images from the femurs and vertebrae with 9‐micron voxel resolution, 50 kV X‐ray voltage, 200 μA current, 0.5 mm aluminum filter, and 1° rotation step. Reconstruction of the images was performed using NRecon (version 1.6.9.18) and DataViewer (version 1.5.1.2). For the trabecular and cortical reconstruction of femurs, the region commenced about 0.215 mm and 1.935 mm from the growth plate in the direction of metaphysis, and extended from this position for a further 1.29 mm and 0.43 mm, respectively. The structural indices were calculated using the Bruker Skyscan CT Analyzer (version 1.14.4.1). In accordance with the ASBMR guidelines for the use of micro‐CT in rodents,^(^ ^51^ ^)^ trabecular and cortical parameters were calculated. The three‐dimensional model of femur (whole, trabecular, and cortical bone) and vertebrae were created using CTVox.

### Histomorphometry

For static histomorphometry, femurs were isolated, fixed in 4% paraformaldehyde for 3 days, decalcified with 15% ethylenediamine tetraacetic acid (EDTA) for 10 days and embedded in paraffin as previously described.^(^ ^52^ ^)^ Histomorphometric analysis of 7‐micron tartrate‐resistant acid phosphatase (TRAP)‐stained sections was carried out according to the standard procedures using the Osteomeasure system (OsteoMetrics, Decatur, GA, USA).^(^ ^53^, ^54^ ^)^ The following parameters were measured: osteoclast surface per unit bone surface (Oc.S/BS), osteoclast number per bone perimeter (Oc.N/B.Pm), osteoblast surface per unit bone surface (Ob.S/BS), and osteoblast number per bone perimeter (Ob.N/B.Pm) as shown in Fig. 4. The analysis was performed with observer blinded to the genotype.

### PINP and CTX‐I ELISAs

Blood was collected in heparin‐coated tubes and centrifuged (2000*g*; 10 minutes; RT) to collect the supernatant (plasma). N‐terminal propeptide of type I procollagen (PINP) (cat. AC‐33F1; Immunodiagnostic Systems, Boldon, UK) and C‐terminal telopeptides of type I collagen (CTX‐I) ELISAs (AC‐06F1; Immunodiagnostic Systems) were performed according to the manufacturer's instructions.

### siRNA transfection

The siGENOME non‐targeting control siRNA pool # 2 (*NT* # 2) (cat. D‐001206‐14‐05) and siGENOME mouse *Shank2* siRNA (cat. M‐060012‐01‐0005) were purchased from Horizon Discovery (PerkinElmer, Waltham, MA, USA). The reverse transfection was performed using a final concentration of 20 nM siRNA with 0.125% lipofectamine RNAiMAX transfection reagent (cat. 13778075; Thermo Fisher Scientific), as previously described.^(^ ^47^ ^)^ The mouse siRNA sequences used are listed in Table 1.

**Table 1:** Mouse siRNA Sequences Used in This Study

Gene symbol	Gene name	Gene ID	Reverse primer (5′–3′)
*NT # 2*	*—*	*—*	*UAAGGCUAUGAAGAGAUAC* *AUGUAUUGGCCUGUAUUAG* *AUGAACGUGAAUUGCUCAA* *UGGUUUACAUGUCGACUAA*
*Shank2*	*SH3 and multiple ankyrin repeat domain 2*	*210274*	*GGAAUCACCUCAUCCUUAA* *GGAAGGGCGUGCUGGUAAA* *CAACGGAAUUGAGCAAAGA* *GGAGGGCGGACGACAAGAA*
*Dlg4*	*Discs large MAGUK scaffold protein 4*	*13385*	*GGGAACAGCUUAUGAAUAG* *GAUCAUCGCUCAGUAUAAA* *GAAGAACACAUAUGACGUU* *GAUAUGAGUUGCAGGUGAA*
*Gkap1*	*G kinase anchoring protein 1*	*56278*	*GAAAUCCUCCCAUUCCAUU* *GGCAAAGGCCGGAGUAAUG* *AGGCGUUGCUGUUGAGUAA* *GCAAACGAGCUCAGGAAUC*
*Plcb3*	*Phospholipase C, beta 3*	*18797*	*GGAGUAAGUUCAUCAAAUG* *GCAGCGAGAUGAUUUGAUU* *GAACCAGGAUGGACGGAUU* *GCAACUAGCCGCUCUCAUU*
*Lrrc7*	*Leucine rich repeat containing 7*	*242274*	*GGACGGUGCUUAAUUCAAA* *GCGCAUGACUGUUGCCUUU* *GUAAGAAUGUCACCGUUAU* *CUAAAGAUGCCGUCCAUAA*
*Cftr*	*Cystic fibrosis transmembrane conductance regulator*	*12638*	*GUACAGAUAUGGUAUGAUU* *UGUCAAAGCUUGCCAACUA* *ACACAUACCUACGAUAUUU* *GCCAUUAGCUCCUCGGAAA*
*Abp1*	*Actin‐binding protein 1*	*13169*	*CGACAUCGACAGUCUAUAA* *GGUAUUGACUGUCCAGAGA* *GCAUGCAGACUAAGUAUAU* *GCGAUGGCCCUGUCCUUUA*
*Cttn*	*Cortactin*	*13043*	*CGAGAGAGCUCAGCGGAUG* *CCAGUAAUAUCCGUGCUAA* *GAAAGAUUACUCCAAAGGU* *CUAUAAGACUGGUUUCGGA*
*Abi1*	*Abl interactor 1*	*11308*	*GGAAACCUAUCGACUAUAC* *CGACACAACUCUACCACUU* *UGCAAGAACUGGCACAUUG* *GGGACUUUGGGACGGAAUA*

### PrestoBlue cell viability assay

The cell viability was determined by using PrestoBlue cell viability reagent (cat. A13261; Thermo Fisher Scientific). Briefly, remove 3.2 mL of media from a 21 cm^2^ culture dish (6 cm) and add 200 μL of PrestoBlue cell viability reagent. In parallel, add 90 μL of culture medium and 10 μL of PrestoBlue cell viability reagent to a few wells in a 96‐well plate that would serve as a blank. Incubate both plates (37°C; 5% CO_2_; 30 minutes). Next, transfer 100 μL from each 21 cm^2^ culture dish into the same 96‐well containing the blank. Measure the absorbance at 570 nm using the Dynex Opsys MR microplate reader (cat. 1MRA‐2497; Aspect Scientific, Tarporley, UK).

### Quantitative and qualitative alkaline phosphatase (Alp) staining

For colorimetric Alp quantification, primary murine osteoblasts isolated from calvaria and long bones were seeded in 21 cm^2^ dishes and 1.9 cm^2^ plate wells, respectively. The cells were differentiated by adding osteogenic induction medium (100 μg/ml (+)‐sodium L‐ascorbate and 5 mM glycerophosphate). The Alp activity was determined by using the Amplite colorimetric alkaline phosphatase assay kit (cat. ABD‐11950; Biomol, Hamburg, Germany) by following the manufacturer's instructions. Briefly, the cells were washed twice with PBS and 2 mL and 300 μL of pNPP solution was added to 21 cm^2^ dishes and 1.9 cm^2^ plate wells, respectively. The plates were incubated (37°C; 1 hour), and the absorbance was measured at 405 nm using the Dynex Opsys MR microplate reader. For quantification, the Alp activity measurements were divided by the cell viability (PrestoBlue staining) measurements. Results were presented as percent change relative to the control, which was set to 100%.

For fluorescence‐based Alp quantification, primary calvarial osteoblasts were reverse transfected and seeded in 0.056 cm^2^ of each well in a 384‐well plate (1.8 × 10^3^ cells/well) and differentiated by adding osteogenic induction medium. The cells were fixed, stained, and analyzed as previously described.^(^ ^47^ ^)^ For quantification, the Alp activity was determined and the cell number was normalized as a percentage of the corresponding average of the non‐targeting (si*NT*) controls.

For qualitative Alp staining, a previously described method was used.^(^ ^47^ ^)^ Briefly, the primary murine osteoblasts isolated from calvaria and long bones were washed twice with PBS, fixed with 4% paraformaldehyde (10 minutes; RT), and stained by adding 2 mL and 300 μL of with Alp solution, respectively. The plates were incubated in the dark for 60 minutes at RT and imaged using Leica microscope (cat. M125; Leica Microsystems, Buffalo Grove, IL, USA).

### Alizarin red S staining and quantification

To determine the mineralization state of primary murine osteoblasts isolated from calvaria and long bones and human iPSCs, we used a previously described method.^(^ ^47^ ^)^ Briefly, the cells were washed twice with PBS and fixed with 4% paraformaldehyde (10 minutes; RT). The nodules were stained with 1% Alizarin red S (cat. A5533; Sigma‐Aldrich) and incubated for 30 to 60 minutes. The cells were washed thrice with PBS and imaged using Leica microscope.

For quantitative staining, 10% acetic acid was added to each 21 cm^2^ dish and 1.9 cm^2^ plate wells, respectively, which were stained with 1% Alizarin red S as described previously.^(^ ^47^ ^)^ The plates were incubated (700 rpm; 30 minutes; RT). Using cell scrapper, the cells were gently removed from the plates and transferred into 1.5 mL tubes, vortexed, and put on the heating block (85°C; 10 minutes). Samples were cooled and centrifuged (211 *g*; 15 minutes; RT). After centrifugation, 100 μL of the supernatant was neutralized with 100 μL of 10% ammonium hydroxide. The absorbance was measured at 405 nm using the Dynex (Chantilly, VA, USA) Opsys MR microplate reader. For quantification, the Alizarin red S staining (OD_405_) was normalized by the absorbance of PrestoBlue staining (OD_570_).

### Ki67 staining of primary osteoblasts isolated from long bones

The Ki67 staining was performed as previously described.^(^ ^47^ ^)^ Briefly, the primary osteoblasts isolated from long bones were plated on 0.32 cm^2^ of each well in a 96‐well plate (6.4 × 10^3^ cells/well). After 48 hours of seeding, the culture medium was replaced with osteogenic induction medium. At day 17, the cells were fixed with 4% paraformaldehyde for 10 minutes, permeabilized with 0.2% Triton X‐100 (cat. X100; Sigma) for 10 minutes, blocked with 30 μL of IHC select blocking buffer (cat. 20773; Merck) for 30 minutes, and stained with rabbit anti‐Ki67 (cat. Ab15580; Abcam, Cambridge, UK) antibody (1:100 in blocking buffer) (1 hour; RT). The cells were washed thrice in PBS and 30 μL of Alexa‐488 anti‐rabbit secondary antibody (1:400 in blocking buffer) (cat. A21206; Thermo Fisher Scientific) was added (45 minutes; RT). The cells were washed again thrice with PBS followed by DAPI staining (1 μg/mL) (cat. 62248; Thermo Fisher Scientific) (5 minutes; RT). The samples were imaged using ImageXpressMicro confocal microscope (Molecular Devices, San Jose, CA, USA)^(^ ^47^ ^)^ and were analyzed using cell profiler software.^(^ ^55^ ^)^

### Colony‐forming unit (CFU) assay

For CFU assay, primary osteoblasts isolated from long bones were plated on a 56 cm^2^ sterile culture dish (1 × 10^3^ cells/dish) and incubated in complete medium (α‐MEM containing 15% FBS; 1% penicillin/streptomycin; 1% L‐glutamine) as described previously.^(^ ^56^ ^)^ At day 17, the cultures were fixed with 100% methanol (10 minutes; RT) and stained with 0.5% Crystal violet (cat. C0775; Sigma) in 25% methanol for 10 minutes. The cells were washed with distilled water and number of colonies were counted under light microscope.

### RNA isolation, cDNA synthesis, and qPCR

Total RNA was extracted from different organs using Trizol reagent (cat. 15596026; Thermo Fisher Scientific) and from cells using RNeasy Kit (cat. 75142; Qiagen, Valencia, CA, USA) according to the manufacturer's instructions. For long bones, all surrounding tissue was cleared, the epiphyses were removed, and bones were centrifuged (14,674 *g*; 1 minute; RT), and total RNA was isolated using Trizol reagent. Reverse transcription of RNA from cells and organs was performed using RevertAid H Minus reverse transcriptase kit (cat. EP0451; Thermo Fisher Scientific) or high‐capacity cDNA kit (cat. 4368814; Thermo Fisher Scientific) with 1000 ng of RNA. qPCR was performed on a ViiA 7 (Applied Biosystems, Carlsbad, CA, USA), and relative mRNA concentrations, which were normalized to β‐actin, were calculated by the ΔΔCt method. The human and mouse primers used in qPCR include HMBS (GeneGlobe ID: QT00014462, Qiagen), BGLAP (GeneGlobe ID: QT00232771, Qiagen), and SHANK2 (GeneGlobe ID: QT00030737, Qiagen) and are also listed in Table 2.

**Table 2:** Oligonucleotide Primer Sequences From Mouse and Humans Used in Real‐Time Polymerase Chain Reaction (RT‐PCR)

Gene symbol	Gene ID	Forward primer (5′–3′)	Reverse primer (5′–3′)
*Mouse*			
*Shank1* (*SAM*)	*243961*	*CTCTAGGGTTCTGGACCAAG*	*AATTTGAGAGCCCGGTC*
*Shank2* (*PDZ*)	*210274*	*GAGGAATTCACACCCACGCCAGCA*	*AAGTCCCCGGTCCTTAGTCC*
*Shank2* (*SAM*)	*210274*	*GACGAAACCAGATGTGGCAGACTG*	*TCTTCCTTCTGAAGGTTTGGC*
*Shank3* (*PDZ*)	*58234*	*CGGGGAGCCAAAGCAGAGACC*	*TCTACAGACTCAAGGTATTGGAG*
*Shank3* (*SAM*)	*58234*	*AGCTCTGGAGCAAGTTCGAT*	*TGCGCCTTCGATCTCATGGT*
*Runx2*	*12393*	*TGTTCTCTGATCGCCTCAGTG*	*CCTGGGATCTGTAATCTGACTCT*
*Sp7*	*170574*	*CCCACCCTTCCCTCACTCAT*	*CCTTGTACCACGAGCCATAGG*
*Alpl*	*11647*	*GCTGATCATTCCCACGTTTT*	*CTGGGCCTGGTAGTTGTTGT*
*Bglap*	*12096*	*TCTGACAAAGCCTTCATGTCCA*	*CGGTCTTCAAGCCATACTGGTC*
*Spp1*	*20750*	*TCACCATTCGGATGAGTCTG*	*ACTTGTGGCTCTGATGTTCC*
*Ibsp*	*15891*	*GTCTTTAAGTACCGGCCACG*	*TGAAGAGTCACTGCCTCCCT*
*Actb*	*11461*	*AGAGGGAAATCGTGCGTGAC*	*CAATAGTGATGACCTGGCCGT*
*Human*			
*RUNX2*	*860*	*CTACCACCCCGCTGTCTTC*	*AAAAAGGGCCCAGTTCTGA*
*SP7*	*121340*	*GGAAGAAGCCCATCCACA*	*AAGCCTTGCCATACACCTTG*
*ALPL*	*249*	*AGAACCCCAAAGGCTTCTTC*	*CTTGGCTTTTCCTTCATGGT*
*SPP1*	*6696*	*CCCACAGACCCTTCCAAGTA*	*ACACTATCACCTCGGCCATC*
*IBSP*	*3381*	*CAACAGCACAGAGGCAGAAA*	*CGTACTCCCCCTCGTATTCA*

### Protein isolation, quantification, and Western blotting

Total cellular protein was extracted using radioimmunoprecipitation assay (RIPA) buffer, quantified by Pierce BCA protein assay kit (cat. 23225; Thermo Fisher Scientific) and subjected to Western blotting as previously described,^(^ ^47^ ^)^ using antibodies against Shank2 (1:500) (cat. ab94575; Abcam), and β‐actin (1:1000) (cat. A1978; Sigma). The band intensity of Western blots was quantified using Fiji ImageJ (v2.3.0).^(^ ^57^ ^)^

### Statistical analysis

Assuming normally distributed data, statistical differences between two groups were determined by unpaired two‐tailed student's *t* test, Welch's *t* test, in case of unequal variances. A *p* value <0.05 was regarded as statistically significant difference, *p < 0.05, **p < 0.01, ***p < 0.001.

## Results

### *Shank2* is expressed in bone tissue, and levels are elevated during osteoblast differentiation

**Fig. 1:**
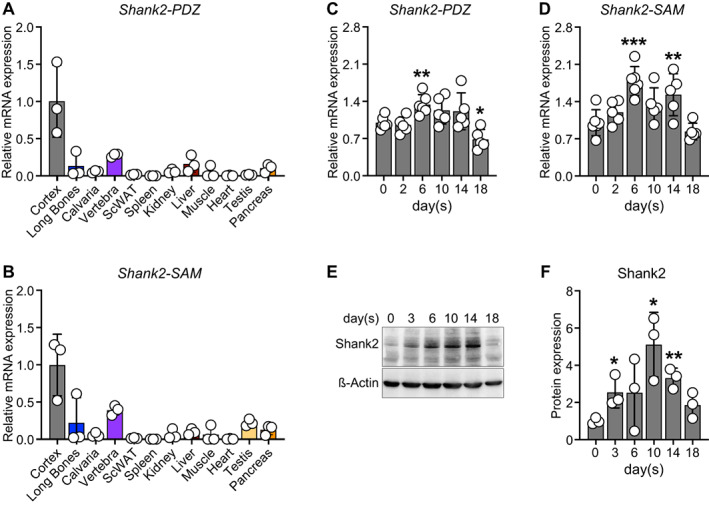
Shank2 is expressed in skeleton and its expression is increased during the course of osteoblast differentiation. (*A, B*) qPCR analysis of *Shank2‐PDZ* and *‐SAM* domain mRNA levels of brain cortex, long bones, calvaria, vertebra, subcutaneous white adipose tissue (ScWAT), spleen, kidney, liver, muscle, heart, testis, and pancreas (*n* = 3), which were isolated from 13‐week‐old male mice. (*C, D*) qPCR analysis of *Shank2‐PDZ* and *‐SAM* domain mRNA levels over the course of osteoblast differentiation in primary murine calvarial osteoblasts (*n* = 5–6). (*E*) Shank2 protein levels by Western blotting and its (*F*) quantification during the course of osteoblast differentiation in MC3T3‐E1 cell line (*n* = 3). Statistical differences between two groups were determined by unpaired two‐tailed Welch's *t* test. *p < 0.05, **p < 0.01, ***p < 0.001.

In the brain, multiple isoforms of Shank2 are expressed, encompassing different domains with distinct functions. In particular, the PDZ domain and the SAM domain are hubs of protein interaction in the synapses. To determine their peripheral expression in bone, we thus tested the expression of mRNA transcripts in long bones, calvaria, and vertebrae and compared mRNA levels with other tissues, including brain cortex. mRNA encoding both domains can be detected most prominent in brain cortex and to a moderate degree in long bones (~7.6‐fold decrease), vertebrae (~3.5‐fold decrease), liver, and pancreas, with low expression in spleen, muscle, and heart (Fig. 1*A, B*). Next, we tested the expression pattern during osteoblast differentiation in primary calvarial osteoblasts. We first analyzed the early‐ (*Runx2, Sp7*, and *Alpl*) and late‐stage (*Bglap, Spp1*, and *Ibsp*) marker gene expression and observed their increased expression during the course of differentiation (Supplemental Fig. S1 *A–F*). In addition, we could detect *Shank2* mRNA coding for the *‐PDZ* and *‐SAM* domain in osteoblasts and found that their levels are also modulated during the course of differentiation (*Shank2‐PDZ*: days 6 and 18; *Shank2‐SAM*: days 6 and 14) (Fig. 1*C, D*), suggesting a pivotal role of *Shank2* during osteoblast differentiation. Moreover, we evaluated the mRNA expression of other members of the Shank family (*Shank1‐SAM*, *Shank3‐PDZ*, and *Shank3‐SAM*) and observed increased expression of *Shank1‐SAM* and *Shank3‐PDZ*, with no change in *Shank3‐SAM* during the course of differentiation (Supplemental Fig. S2 *A–C*). Similar to the mRNA expression in primary calvarial osteoblasts, the Shank2 protein concentrations are also elevated (days 3, 10, and 14) in MC3T3‐E1 osteoblastic cell line (Fig. 1*E, F*). Taken together, we show that *Shank2* is expressed within the bone tissue and in bone‐forming osteoblasts.

### Deletion of *Shank2* by siRNA reduces osteoblast differentiation and mineralization

**Fig. 2:**
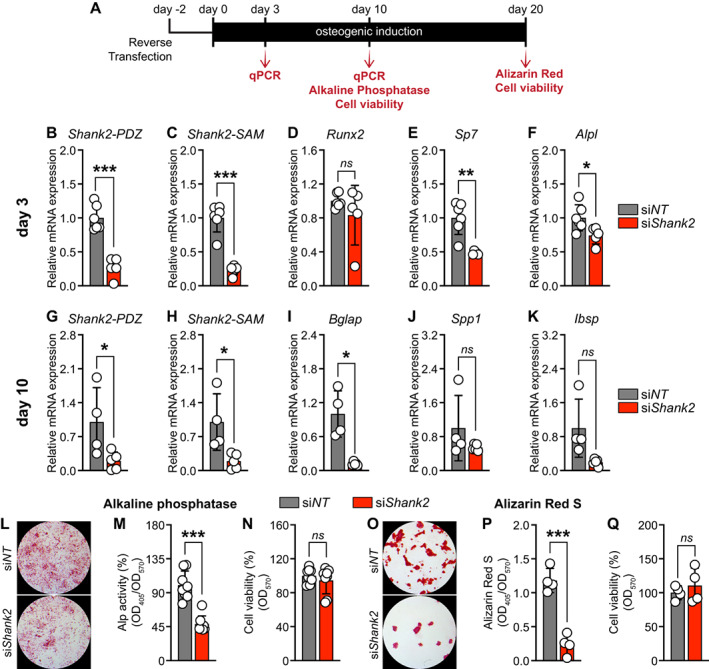
Silencing of *Shank2* decreases osteoblast differentiation and mineralization in primary murine calvarial osteoblasts. (*A*) Scheme showing siRNA reverse transfection strategy and assays carried out at different time points during osteoblast differentiation. qPCR analysis of (*B–F*) *Shank2‐PDZ*, *Shank2‐SAM*, and early‐stage (*Runx2, Sp7, Alpl*) (*n* = 5–6) osteoblast‐specific marker gene expression from si*NT* or si*Shank2* after 5 days of transfection in primary murine calvarial osteoblasts. qPCR analysis of (*G–K*) *Shank2‐PDZ*, *Shank2‐SAM*, and late‐stage (*Bglap, Ibsp, Spp1*) (*n* = 4–5) osteoblast‐specific marker gene expression from si*NT* or si*Shank2* after 12 days of transfection in primary murine calvarial osteoblasts. (*L, M*) Qualitative and quantitative Alp staining, and (*N*) cell viability assay using PrestoBlue staining in primary murine calvarial osteoblasts transfected with si*NT* or si*Shank2* after 12 days of transfection (*n* = 8). (*O, P*) Qualitative and quantitative Alizarin red S staining by acetic acid extraction method, and (*Q*) cell viability assay using PrestoBlue staining in primary murine calvarial osteoblasts transfected with si*NT* or si*Shank2* after 22 days of transfection (*n* = 4). Statistical differences between two groups were determined by unpaired two‐tailed Welch's *t* test. *p < 0.05, **p < 0.01, ***p < 0.001.

The early increased expression during osteoblast differentiation suggests a functional role of Shank2 in the initial phase of osteoblast differentiation. We therefore silenced *Shank2* by transfecting primary murine calvarial osteoblasts with *Shank2*‐specific siRNA (si*Shank2*), which reduced the expression of transcripts coding for *Shank2‐PDZ* and *‐SAM* domain at day 3 (Fig. 2*A–C*). This was accompanied by a reduced expression of marker genes for early osteoblast differentiation such as *Sp7* and *Alpl*, without affecting the *Runx2* expression (Fig. 2*D–F*). Importantly, the decreased osteoblast differentiation persisted also at later stages of osteoblast differentiation. At day 10, the expression of transcripts coding for *Shank2‐PDZ* and *‐SAM* domain as well as the late osteoblast marker gene *Bglap* were also decreased, without affecting the *Spp1* and *Ibsp* expression (Fig. 2*G–K*). Moreover, *Shank2* knockdown by siRNA (si*Shank2*) markedly decreased qualitative and quantitative alkaline phosphatase (Alp) activity when compared with non‐targeting siRNA (si*NT*) control, without affecting the cell viability (Fig. 2*L–N*). Furthermore, we showed a strong decrease in bone nodule formation at day 20 in si*Shank2* when compared with the si*NT*, as determined by Alizarin red S staining and quantification, without impacting cell viability (Fig. 2*O–Q*). Taken together, we show that osteoblast differentiation and mineralization capacity of primary calvarial osteoblasts is reduced upon *Shank2* depletion.

### *Shank2* knockout male mice exhibit reduced bone mass due to decrease in osteoblastogenesis

**Fig. 3:**
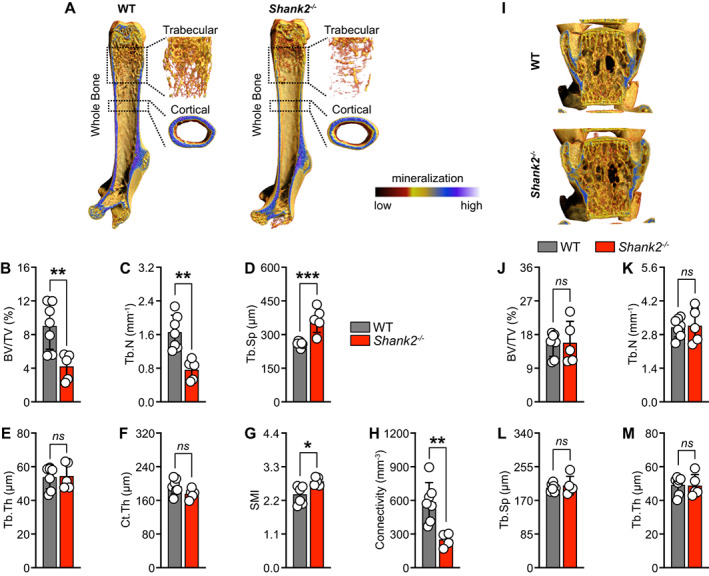
*Shank2* ^*−/−*^ mice exhibit decrease in bone mass. (*A*) Representative micro‐computed tomography (micro‐CT) images of whole, trabecular, and cortical bone of femurs isolated from 13‐week‐old, male wild‐type (WT) and Shank2 knockout (*Shank2* ^*−/−*^) mice. Calculated trabecular and cortical parameters of femurs from WT and *Shank2* ^−/−^ mice that include: (*B*) percent bone volume (BV/TV; %), (*C*) trabecular number (Tb.N; mm^−1^), (*D*) trabecular separation (Tb.Sp; μm), (*E*) trabecular thickness (Tb.Th; μm), (*F*) cortical thickness (Ct.Th; μm), (*G*) structure model index (SMI), and (*H*) connectivity (mm^−3^) (*n* = 5–7). (*I*) Representative micro‐CT images of vertebra isolated from 13‐week‐old, male WT and *Shank2* ^*−/−*^ mice. Calculated trabecular parameters of vertebra from WT and *Shank2* ^−/−^ mice that include: (*J*) BV/TV (%), (*K*) Tb.N (mm^−1^), (*L*) Tb.Sp (μm), and (*M*) Tb.Th (μm). Statistical differences between two groups were determined by unpaired two‐tailed Student's *t* test. *p < 0.05, **p < 0.01, ***p < 0.001.

To examine the in vivo effects of *Shank2* on skeletal homeostasis, we analyzed *Shank2* knockout (*Shank2* ^*−/−*^) male mice for their bone mass. Micro‐computed tomography (micro‐CT) analysis in the distal femurs of *Shank2* ^*−/−*^ mice exhibit a decrease in trabecular bone volume (BV/TV), trabecular number (Tb.N), and subsequently an increase in trabecular separation (Tb.Sp) (Fig. 3*A–D*). No significant differences were observed in trabecular thickness (Tb.Th) and cortical thickness (Ct.Th) in *Shank2* ^*−/−*^ mice when compared with the WT mice (Fig. 3*A, E, F*). In addition, we observed significant increases in structure model index (SMI) and decrease in connectivity in *Shank2* ^*−/−*^ mice when compared with the WT controls (Fig. 3*G, H*). However, no significant changes were observed in BV/TV, Tb.N, Tb.Sp, and Tb.Th in vertebrae (Fig. 3*I–M*). Thus, the absence of *Shank2* strongly decreased trabecular bone mass in femur, without affecting the bone structural phenotype in vertebrae.

**Fig. 4:**
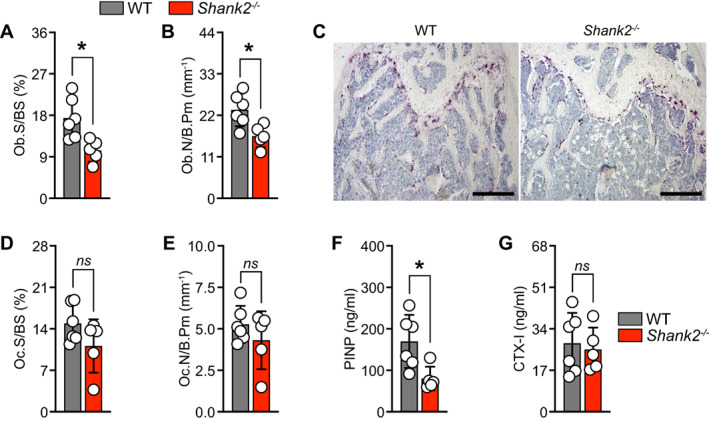
Analysis of *Shank2* ^*−/−*^ mice shows decrease in osteoblastogenesis. Bone histomorphometry was performed in femurs from wild‐type (WT) and *Shank2* ^−/−^ mice and following parameters were calculated from trabecular bone: (*A*) Percent osteoblast surface per bone surface (Ob.S/BS; %), (*B*) osteoblast number per bone perimeter (Ob.N/B.Pm; mm^−1^). (*C*) Representative images of tartrate‐resistant acid phosphatase (TRAP) staining for osteoclasts (purple) (scale bar = 500 μm). (*D*) Osteoclast surface per bone surface (Oc.S/BS; %), and (*E*) osteoclast number per bone perimeter (Oc.N/B.Pm; mm^−1^) (*n* = 5–6). Analysis of bone formation marker and bone resorption markers (*F*) N‐terminal propeptide of type I procollagen (PINP) (ng/mL), and (*G*) C‐terminal telopeptides of type I collagen (CTX‐I) (ng/mL) (*n* = 5–6). Statistical differences between groups were determined by unpaired two‐tailed Student's *t* test. *p < 0.05.

Bone histomorphometry revealed a strong reduction in osteoblast surface per bone surface (Ob.S/BS) and osteoblast number per bone perimeter (Ob.N/B.Pm) (Fig. 4*A, B*). No changes in the osteoclast surface per bone surface (Oc.S/BS) and osteoclast number per bone perimeter (Oc.N/B.Pm) were observed in *Shank2* ^*−/−*^ mice when compared with the WT control mice (Fig. 4*C–E*). Accordingly, in blood plasma, the bone formation marker (PINP) level was significantly reduced, whereas no changes in the level of bone resorption marker (CTX‐I) was observed in *Shank2* ^*−/−*^ mice when compared with the WT littermates (Fig. 4*F, G*).

Taken together, we showed that *Shank2* ^*−/−*^ mice exhibited reduced bone mass compared with their WT control littermates, which is most likely due to a defective osteoblastogenesis.

### Primary *Shank2^‐/‐^* osteoblasts show decreased mineralization

**Fig. 5:**
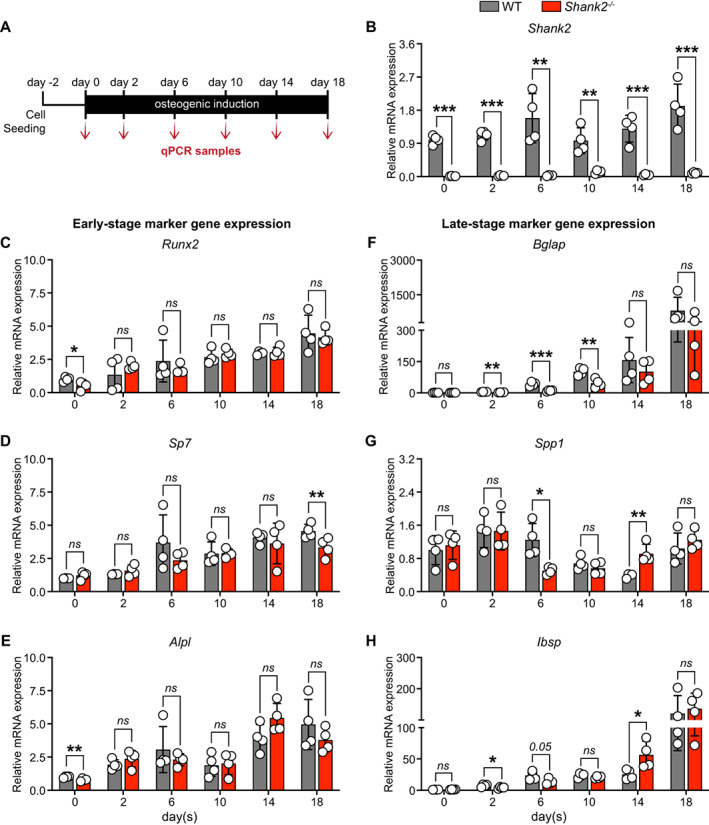
Primary osteoblasts isolated from *Shank2* ^*−/−*^ long bones shows that osteoblast‐specific marker gene expression remains unaffected. (*A*) Experimental setup. qPCR analysis of: (*B*) *Shank2* expression, (*C–E*) early‐stage (*Runx2, Sp7, Alpl*), and (*F–H*) late‐stage (*Bglap, Ibsp, Spp1*) osteoblast‐specific marker gene expression over the course of osteoblast differentiation in primary osteoblasts isolated from wild‐type (WT) and *Shank2* ^*−/−*^ long bones (*n* = 4). Statistical differences between groups were determined by unpaired two‐tailed Student's *t* test. *p < 0.05, **p < 0.01, ***p < 0.001.

**Fig. 6:**
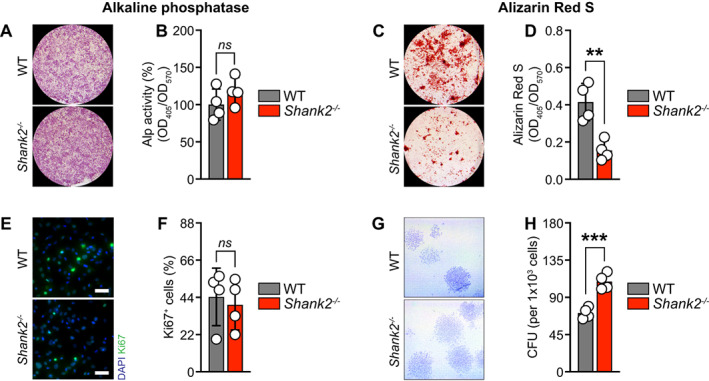
Primary osteoblasts isolated from *Shank2* ^−/−^ long bones shows decreased mineralization. (*A, B*) Qualitative and quantitative Alp staining after 15 days of osteogenic induction in primary osteoblasts isolated from wild‐type (WT) and *Shank2* ^−/−^ long bones (*n* = 4). (*C*) Qualitative, and (*D*) quantitative Alizarin red S staining by acetic acid extraction method in primary osteoblasts isolated from WT and *Shank2* ^−/−^ long bones after 36 days of osteogenic induction (*n* = 4). (*E*) Representative pictures of Ki67 staining (nuclei: blue; Ki67^+^: green), and (*F*) its quantification after 15 days of osteogenic induction in primary osteoblasts isolated from WT and *Shank2* ^−/−^ long bones (*n* = 4). (*G*) Representative pictures of crystal violet staining showing colony‐forming unit (CFU) per 1000 cells seeded in 10‐cm culture dishes (surface area: 56 cm^2^) and (*H*) their quantification after 17 days of seeding in primary osteoblasts isolated from WT and *Shank2* ^−/−^ long bones (*n* = 4). Statistical differences between groups were determined by unpaired two‐tailed Student's *t* test. **p < 0.01, ***p < 0.001.

To investigate the intrinsic role of *Shank2* on osteoblast differentiation and mineralization, primary osteoblasts from long bones were isolated from WT and *Shank2* ^*−/−*^ mice. First, deletion of S*hank2* was observed during the whole course of osteoblast differentiation (Fig. 5*A, B*). The expression of early‐stage (*Runx2, Sp7*, and *Alpl*) and late‐stage (*Spp1* and *Ibsp*) osteoblast‐specific marker gene expression remains mostly unaffected (Fig. 5*C–H*). Despite no changes in qualitative and quantitative ALP activity (Fig. 6*A, B*), a significant reduction of bone nodule formation, both qualitative and quantitative, was observed in *Shank2* ^*−/−*^ cells compared with their WT controls (Fig. 6*C, D*). Moreover, the cellular proliferation (indicated by Ki67 staining) remain unaffected, whereas the colony formation potential was significantly increased in *Shank2* ^*−/−*^ cells (Fig. 6*E–H*), suggesting *Shank2* deficiency may promote precursors into osteoblast lineage. Taken together, we showed that primary osteoblasts isolated from *Shank2* ^*−/−*^ mice displayed decreased mineralization.

### Loss‐of‐function mutation in *SHANK2* gene in human iPSCs decreases osteoblast differentiation

**Fig. 7:**
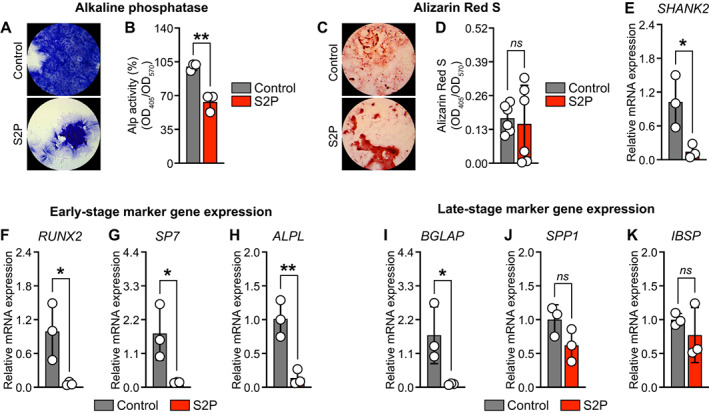
Loss‐of‐function mutation in *SHANK2* gene in human induced pluripotent stem cells (iPSCs) decreases osteoblast differentiation. (*A, B*) Qualitative and quantitative Alp staining in human iPSCs isolated from a healthy patient (Control) or from a patient with loss‐of‐function mutation in *SHANK2* (shown as S2P) gene after 23 days of culture (*n* = 3). (*C*) Qualitative, and (*D*) quantitative Alizarin red S staining by acetic acid extraction method in human iPSCs isolated from control or S2P patient (*n* = 6). (*E*) *SHANK2* expression in human iPSCs isolated from control or S2P patient after 23 days of osteogenic induction, quantified by qPCR analysis (*n* = 3). qPCR analysis of: (*F–H*) early‐stage (*RUNX2, SP7, ALPL*) and (*I–K*) late‐stage (*BGLAP, IBSP, SPP1*) osteoblast‐specific marker gene expression from human iPSCs isolated from control or S2P patient after 23 days of osteogenic induction (*n* = 3). Statistical differences between groups were determined by unpaired two‐tailed Student's *t* test. *p < 0.05, **p < 0.01.

After demonstrating that the decreased *Shank2* expression in mice leads to impaired osteoblastogenesis observed in vitro and diminished osteoblast numbers in vivo with a reduced bone mass, we explored whether this also holds true for human osteoblasts. To investigate the effects of *SHANK2* on osteoblast differentiation in vitro, we used iPSCs from a patient who has a loss‐of‐function mutation in the *SHANK2* gene (hereafter called as S2P).^(^ ^17^ ^)^ Consistent with the findings from murine primary osteoblasts, we observed a marked decrease in qualitative and quantitative Alp activity in S2P when compared with iPSC‐derived osteoblasts from a control patient (Fig. 7*A, B*). However, we did not see any differences in bone nodule formation in S2P when compared with the control, as determined by Alizarin red S staining and quantification (Fig. 7*C, D*). To assess the changes in early‐ and late‐stage osteoblast‐specific marker gene expression, we first confirmed the *SHANK2* downregulation in iPSCs isolated from the S2P patient (Fig. 7*E*). Besides, we observed a marked reduction of early‐stage (*RUNX2*, *SP7*, and *ALPL*) and late‐stage (*BGLAP*) osteoblast‐specific marker gene expression in S2P compared with the iPSCs from the control patient (Fig. 7*F–K*). Taken together, we show that the osteoblast differentiation capacity of human iPSCs is affected by a loss‐of‐function mutation in the human *SHANK2* gene.

### Silencing of Shank2 interacting proteins affect early osteoblast differentiation

**Fig. 8:**
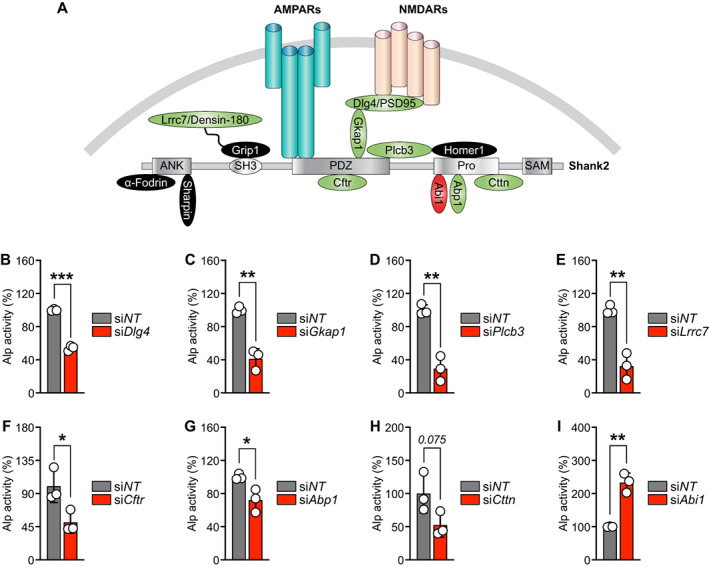
Silencing of Shank2 interacting proteins modulate osteoblast differentiation. (*A*) Schema of the partial Shank2 protein interactome at the postsynaptic density (PSD). The green‐colored proteins reduced, whereas the red‐colored protein enhanced the Alp activity after siRNA knockdown. Cellular Alp activity after 8 days siRNA transfection of: (*B*) discs large MAGUK scaffold protein 4 (*Dlg4*), (*C*) G kinase anchoring protein 1 (*Gkap1*), (*D*) phospholipase C, beta 3 (*Plcb3*), (*E*) leucine rich repeat containing 7 (*Lrrc7*), (*F*) cystic fibrosis transmembrane conductance regulator (*Cftr*), (*G*) actin‐binding protein 1 (*Abp1*), (*H*) cortactin (*Cttn*), and (*I*) Abl interactor 1 (*Abi1*), in primary murine calvarial osteoblasts (*n* = 3). Statistical differences between groups were determined by unpaired two‐tailed Student's *t* test. *p < 0.05, **p < 0.01, ***p < 0.001.

In synapses, Shank2 is able to build the large multimeric complexes via different domains, thereby creating a postsynaptic platform that is used for multiple protein–protein interactions (Fig. 8*A*).^(^ ^21^ ^)^ To test whether Shank2 interacting factors were functionally involved in early osteoblast differentiation, we used specific siRNAs against known Shank interacting proteins (Fig. 8*A*). Interestingly, siRNAs against discs large MAGUK scaffold protein 4 (*Dlg4*), G kinase anchoring protein 1 (*Gkap1*), phospholipase C, beta 3 (*Plcb3*), leucine rich repeat containing 7 (*Lrrc7*), cystic fibrosis transmembrane conductance regulator (*Cftr*), actin‐binding protein 1 (*Abp1*), and Cortactin (*Cttn*) revealed a diminished cellular Alp activity in primary murine calvarial osteoblasts with the exception of Abl interactor 1 (*Abi1*) (Fig. 6*B–I*). These findings suggest that a Shank2 multimeric complex, generally known to be involved in synapse morphology and function, plays also a vital role in regulating osteoblast differentiation.

## Discussion

Here, we describe a novel role of Shank2, a neuronal scaffolding protein. Shank2 is a positive regulator of osteoblast differentiation and bone formation, therefore regulating an important non‐neuronal biological process.

First, we showed that *Shank2* mRNA is well detectable in different bones considerably higher than in other organs, except brain and liver. We also discriminated between mRNAs coding for the *Shank2‐PDZ* domain and/or the *‐SAM* domain. Both of these domains are important for protein–protein interaction in various isoforms of Shank2 known from isoform analysis in the central nervous system.^(^ ^21^ ^)^ Furthermore, our study revealed that Shank2 expression is increased at certain time points, more pronounced at protein than mRNA level during the course of osteoblast differentiation, consistent with the previous findings with osteoblast‐promoting genes that display a similar pattern of expression such as *Runx2*, *Sp7*, and *Bglap*.^(^ ^47^, ^58^ ^)^ Moreover, we demonstrated that *Shank2* depletion reduces the expression of zinc finger‐containing transcriptional factor, *Sp7*, which is known to be indispensable for bone development as shown in *Sp7*‐null mice, in which no cortical or trabecular bone develops through endochondral or intramembranous ossification.^(^ ^59^ ^)^ These data are supported by experiments done with human iPSCs carrying a loss‐of‐function mutation of *SHANK2* where a reduced expression of *RUNX2*, a master regulator of osteoblast differentiation, was detected.^(^ ^60^, ^61^, ^62^ ^)^ However, the limitation of iPSC data from only one patient per group needs further support by increasing the patient cohort. We further demonstrated that *Shank2* reduction also decreases bone mineralization in murine primary osteoblasts isolated from calvaria and long bones. All these findings demonstrate a positive function of *Shank2* in the process of mineralization in vitro.

Mutations within all SHANK genes are known to be causative for ASDs.^(^ ^17^ ^)^ Lower BMD and increased risk of fractures have been observed in both children and young adults with ASD; however, whether this is due to a direct influence of Shank on bone cells was unclear.^(^ ^10^, ^11^, ^12^ ^)^ Previously, we reported that genetic deletion of *Shank2* (*Shank2* ^*−/−*^) in mice leads to autistic‐like behavior and hyperactivity.^(^ ^36^ ^)^ The results of this study show that these *Shank2* ^*−/−*^ mice also exhibit impaired bone homeostasis at least in male mice, consistent with the data observed in human ASD patients.^(^ ^17^ ^)^ We could demonstrate that bone formation is reduced based on undersupply of osteoblasts and reduced bone formation marker, PINP. Osteoclastogenesis and bone resorption marker, CTX‐I seem largely unaffected in *Shank2* ^−/−^ mice, but we cannot rule out a minor effect due to the sample size used. These findings were supported by the reduction of bone mineralization in primary osteoblasts derived from WT and *Shank2* ^*−/−*^ long bones. Shank2 deletion did not affect cellular proliferation but increased the clonal potentiality, suggesting the role of Shank2 in driving the cells toward differentiation by evading clonal expansion. Furthermore, similar findings were observed in osteoblasts derived from iPSCs from a patient with Shank2 mutation.^(^ ^17^ ^)^ Because we investigated global *Shank2*‐deficient animals, we cannot rule out a contribution of neuronal defects on bone mass. However, because of the defective osteoblastogenesis, these results suggest that the reduced BMD observed in Shank2 ASD patients could be in part attributable to the loss‐of‐function mutation in *Shank2* gene in bone.

Strikingly, we did not find changes in trabecular bone volume in the axial skeleton. This could be explained by site‐specific changes in the bone mass in mice, which is mainly due to different precursor cell origin of the axial and appendicular skeleton and different biomechanical loading conditions.^(^ ^63^, ^64^ ^)^ Moreover, to our knowledge, there are no SHANK2 patient studies existing concerning the bone densities in axial skeleton. Thus, further studies need to be performed with patients having SHANK2 mutation and their association with lower BMD to support this hypothesis.

In postsynaptic densities, Shank2 serves as an organizer of large protein complexes that connects different types of glutamate receptors (N‐methyl‐D‐aspartate receptor [NMDAR], α‐amina‐3‐hydroxy‐5‐methyl‐4‐isoxazolepropionic acid receptor [AMPAR], metabotropic glutamate receptor) to molecules of different signaling pathways (Fig. 6*A*).^(^ ^65^ ^)^ Interestingly, in osteoblasts, the treatment with antagonists to NMDA receptors like MK‐801 inhibits ALP activity and downregulates the expression of *Runx2* and *Bglap*.^(^ ^66^, ^67^ ^)^ In contrast, the agonists like NMDA are shown to upregulate the *Bglap* expression and mineralization of osteoblasts.^(^ ^68^ ^)^ In this study, we also observed decreased mineralization in *Shank2*‐depleted primary osteoblasts isolated from calvaria and long bones. However, in contrast to the knockdown experiments by siRNA in calvarial cells, *Shank2* ^*−/−*^ cells showed mostly no differences in osteoblast marker gene expression and Alp staining but prominent reduction in mineralization. These differences could be due to the fact that siRNA affects other *Shank2* isoforms than the knockout of the exon 7 in the *Shank2* ^*−/−*^ cells. This requires further analysis. Despite not finding a difference in osteoclast numbers or resorptive activity, we cannot entirely exclude it because of the sample size used. Glutamate has also been reported in osteoclast and osteocyte function, which could be altered by Shank2 mutation.^(^ ^69^, ^70^, ^71^ ^)^ Furthermore, loss‐of‐function of *Sharpin*, an interacting partner of the *Shank* family via the ankyrin (ANK) domain (Fig. 6*A*), induces to the development of chronic proliferative dermatitis mutations (*Cpdm*) and an osteopenic phenotype in mice.^(^ ^72^ ^)^ This phenotype was solely explained by impaired bone formation without affecting osteoclastogenesis.^(^ ^73^ ^)^ We observed a similar phenotype in the *Shank2* ^*−/−*^ mice due to decrease in osteoblastogenesis, whereas osteoclastogenesis remains unaffected. Because *Sharpin* is a downstream target of *Shank2*, it is reasonable to anticipate that *Shank2* might exert these phenotypic effects through altered *Sharpin* complexes.

Most strikingly, our study identified that silencing of Shank2 interacting proteins, which are downstream targets of NMDAR and AMPAR signaling such as *Dlg4*, *Gkap1*, *Plcb3*, *Lrrc7*, *Cftr*, *Abp1*, and *Cttn*, reduced osteoblastogenesis. These proteins interact directly or indirectly with the PDZ domain, SH3 domain, or proline‐rich region of Shank2, an important structural hub in the postsynaptic density.^(^ ^21^ ^)^ One exception was the downregulation of *Abi1*. Previously, it was suggested in nerve cells that Abi1 dissociates from the complex after NMDA signaling and translocates into the nucleus to interfere with Myc/Max transcription factors.^(^ ^74^ ^)^ Whether this leads to parallel transcriptional programs interfering with osteoblast differentiation needs further investigations. For most of these interacting partners, it is not known whether they regulate osteoblast differentiation.

Taken together, our findings suggest that *Shank2* and its putative interacting factors play an important function by affecting osteoblastogenesis and might present a hitherto not identified network of osteoblast regulators.

## Conflicts of Interests

The authors declare that they have no competing interests.

## Author Contributions

**Mubashir Ahmad:** Data curation; investigation; methodology; writing – original draft. **Nadine Stirmlinger:** Investigation; methodology. **Irfana Jan:** Formal analysis; investigation. **Ulrich Stifel:** Investigation. **Sooyeon Lee:** Data curation; investigation. **Marcel Weingandt:** Investigation. **Ulrike Kelp:** Investigation; methodology. **Jürgen Bockmann:** Supervision. **Anita Ignatius:** Supervision. **Tobias M. Böckers:** Conceptualization; resources; writing – review and editing. **Jan Tuckermann:** Conceptualization; resources; writing – review and editing.

### Peer Review

The peer review history for this article is available at https://publons.com/publon/10.1002/jbm4.10711.

## Supporting information


**Fig. S1.** Early‐ and late‐stage osteoblast‐specific marker gene expression is increased during the course of osteoblast differentiation. qPCR analysis of: (A‐C) early‐stage (*Runx2, Sp7, Alpl*), and (D‐F) late‐stage (*Bglap, Spp1, Ibsp*) osteoblast‐specific marker gene expression during the course of osteoblast differentiation in primary murine calvarial osteoblasts (n = 5‐6).

**Fig. S2.** *Shank1* and *Shank3* expression during the course of osteoblast differentiation. qPCR analysis of: (A) *Shank1‐SAM*, and *Shank3‐PDZ* and ‐*SAM* domain during the course of osteoblast differentiation in primary murine calvarial osteoblasts (n = 5‐6).
