# Supplementary material for: Downregulation of the Autism Spectrum Disorder Gene Shank2 Decreases Bone Mass in Male Mice
Source: JBMR Plus. 2022 Dec 15;7(2):e10711. doi: 10.1002/jbm4.10711 (PMC9893268; doi:10.1002/jbm4.10711)
Supplement: Supplementary file 1 — Fig. S1. Early‐ and late‐stage osteoblast‐specific marker gene expression is increased during the course of osteoblast differentiation. qPCR analysis of: (A‐C) early‐stage (Runx2, Sp7, Alpl), and (D‐F) late‐stage (Bglap, Spp1, Ibsp) osteoblast‐specific marker gene expression during the course of osteoblast differentiation in primary murine calvarial osteoblasts (n = 5‐6). [file JBM4-7-e10711-s001.pdf]

Supplementary figure 1

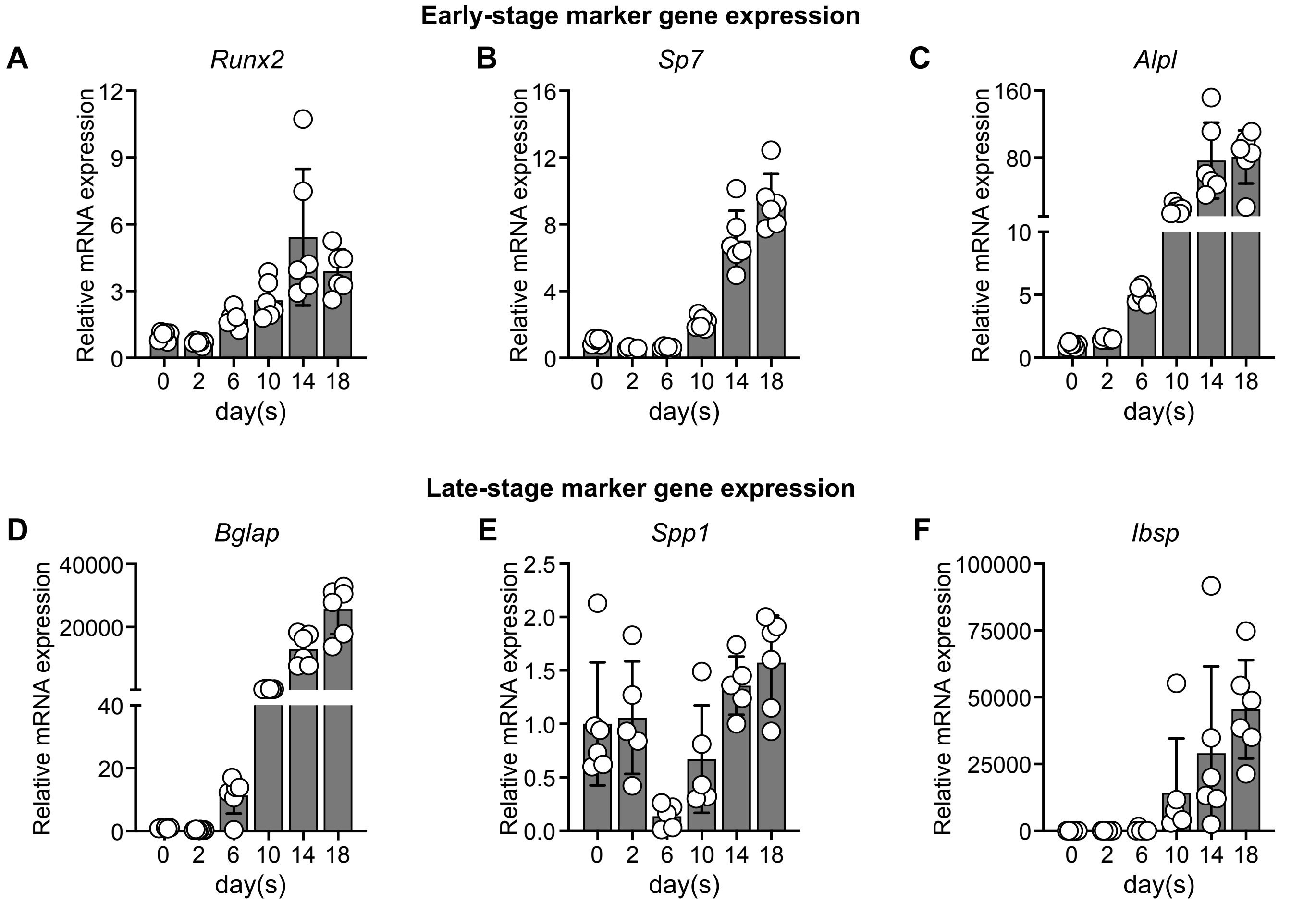

Supplementary Fig. 1 (S1). Early- and late-stage osteoblast-specific marker gene expression is increased during the course of osteoblast differentiation. qPCR analysis of: (A-C) early-stage (Runx2, Sp7, Alpl), and (D-F) late-stage (Bglap, Spp1, Ibsp) osteoblast-specific marker gene expression during the course of osteoblast differentiation in primary murine calvarial osteoblasts (n=5-6).
