# Supplementary material for: Downregulation of the Autism Spectrum Disorder Gene Shank2 Decreases Bone Mass in Male Mice
Source: JBMR Plus. 2022 Dec 15;7(2):e10711. doi: 10.1002/jbm4.10711 (PMC9893268; doi:10.1002/jbm4.10711)
Supplement: Supplementary file 2 — Fig. S2. Shank1 and Shank3 expression during the course of osteoblast differentiation. qPCR analysis of: (A) Shank1‐SAM, and Shank3‐PDZ and ‐SAM domain during the course of osteoblast differentiation in primary murine calvarial osteoblasts (n = 5‐6). [file JBM4-7-e10711-s002.pdf]

Supplementary figure 2

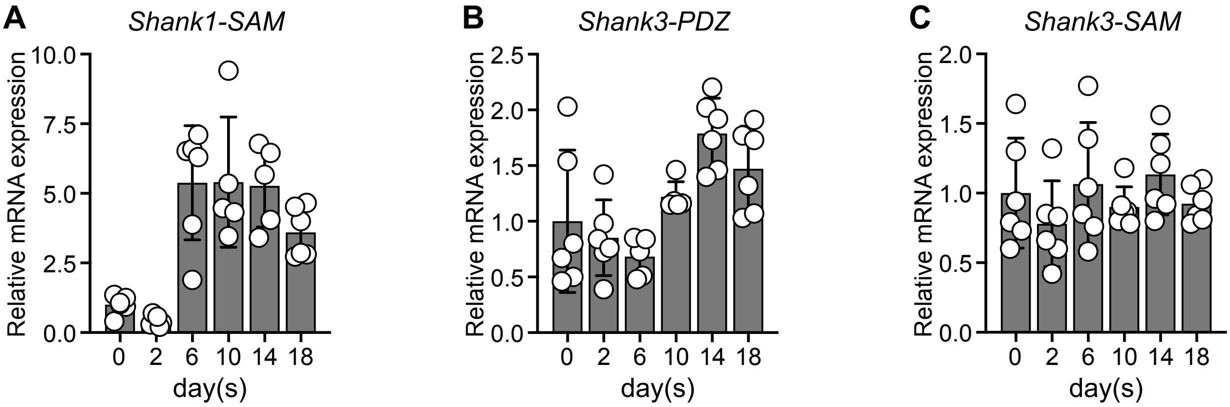

Supplementary Fig. 2 (S2). Shank1 and Shank3 expression during the course of osteoblast differentiation. qPCR analysis of: (A) Shank1-SAM, and Shank3-PDZ and -SAMdomain during the course of osteoblast differentiation in primary murine calvarial osteoblasts (n=5-6).
